# Transcriptional Basis for the Inhibition of Neural Stem Cell Proliferation and Migration by the TGFβ-Family Member GDF11

**DOI:** 10.1371/journal.pone.0078478

**Published:** 2013-11-07

**Authors:** Gareth Williams, Marc P. Zentar, Sangeetha Gajendra, Martina Sonego, Patrick Doherty, Giovanna Lalli

**Affiliations:** Wolfson Centre for Age-Related Diseases, King’s College London, Guy’s Campus, London, United Kingdom; UAE University, Faculty of Medicine & Health Sciences, United Arab Emirates

## Abstract

Signalling through EGF, FGF and endocannabinoid (eCB) receptors promotes adult neurogenesis, and this can be modelled in culture using the Cor-1 neural stem cell line. In the present study we show that Cor-1 cells express a TGFβ receptor complex composed of the ActRIIB/ALK5 subunits and that a natural ligand for this receptor complex, GDF11, activates the canonical Smad2/3 signalling cascade and significantly alters the expression of ∼4700 gene transcripts within a few hours of treatment. Many of the transcripts regulated by GDF11 are also regulated by the EGF, FGF and eCB receptors and by the MAPK pathway – however, in general in the opposite direction. This can be explained to some extent by the observation that GDF11 inhibits expression of, and signalling through, the EGF receptor. GDF11 regulates expression of numerous cell-cycle genes and suppresses Cor-1 cell proliferation; interestingly we found down-regulation of Cyclin D2 rather than p27kip1 to be a good molecular correlate of this. GDF11 also inhibited the expression of numerous genes linked to cytoskeletal regulation including Fascin and LIM and SH3 domain protein 1 (LASP1) and this was associated with an inhibition of Cor-1 cell migration in a scratch wound assay. These data demonstrate GDF11 to be a master regulator of neural stem cell transcription that can suppress cell proliferation and migration by regulating the expression of numerous genes involved in both these processes, and by suppressing transcriptional responses to factors that normally promote proliferation and/or migration.

## Introduction

The final number of cells in a tissue can be limited by the action of polypeptides that inhibit the expansion of progenitor cell populations [1]. The therapeutic importance of this area is well illustrated by the example of the regulation of muscle mass by the TGFβ superfamily member growth and differentiation factor 8 (GDF8, also known as myostatin) [2] that, among other effects, inhibits proliferation of muscle satellite cells during development [3]. In this context, a wide range of GDF8 inhibitors are currently being investigated as therapeutic agents given their ability to promote muscle growth and regeneration [4]–[6]. There is emerging evidence that the almost identical GDF11 (at the level of the mature peptide) can act in an analogous manner to determine neuronal numbers in the brain by regulating neural progenitor proliferation [7]–[9].

GDF8 and GDF11 signal via type I and II TGFβ superfamily receptors, and more specifically bind to activin type II receptors ActRIIA or ActRIIB generally in partnership with the type I activin-like kinase 5 (ALK5) [10]. These receptors can inhibit neurogenesis by controlling expression of p27kip1, a cell-cycle regulatory protein that interacts with cyclin-dependent kinases [7], or progenitor competence via regulation of the transcription factor Math5 [11]. In the present study we have taken a global transcriptional approach and asked how GDF11 signalling integrates with signals from growth promoting receptors to regulate neural stem cell (NSC) function.

The Cor-1 NSC line, derived from E16.5 mouse cortex, can be differentiated to neurons, astrocytes or oligodendrocytes [12]–[14], and relies on EGF, FGF and endocannabinoid (eCB) signalling for optimal proliferation [12], [15], [16]. Diacylglycerol lipase (DAGL) – dependent eCB signalling also regulates the migration of Cor-1 cells (Oudin et al 2011). EGF and FGF receptors are expressed by the rapidly proliferating NSCs in the adult brain [17]–[19] and there is a substantial reduction in proliferation of these cells when their ligands are deleted from the genome [20], [21]. Conversely, the infusion of FGF2 or EGF into the aged mouse brain promotes NSC proliferation [22]. In addition, DAGL-dependent eCB signalling operating via the CB1 and CB2 cannabinoid receptors is required not only for optimal NSC proliferation in both the adult hippocampus and the lateral ventricle subventricular zone (SVZ), but also for the migration of SVZ-derived neuroblasts [23], [24]. Thus, Cor-1 cells are responsive to the same key factors that govern adult neurogenesis and are advantageous for mechanistic studies as they can be grown as a highly homogeneous cell population for biochemical and transcriptional profiling studies [25].

The transcriptional signatures for the EGF, FGF and the CB1 and CB2 cannabinoid receptors, as well as that for the MAPK pathway, have recently become available for Cor-1 cells [25]. In this context, when the cells are grown under optimal conditions in media containing saturating levels of EGF and FGF2 (full media), significant changes are seen in ∼3500 transcripts when the EGF receptor is inhibited. Changes in several hundred transcripts, many of which are co-regulated by the EGF receptor, are seen following inhibition of FGF or eCB receptors. In the present study we show that Cor-1 cells express functional receptors for GDF11, and that treatment of cells grown in full media with this factor significantly alters the expression of ∼4700 transcripts within a few hours. Many of these transcripts are also regulated by the EGF and FGF receptors, with Pearson analysis demonstrating that GDF11 suppresses the responses to these growth factors. We show that this can be explained in part by GDF11 inhibiting the expression of, and downstream signalling from, the EGF receptor. GDF11 also regulated the expression of numerous cell-cycle genes and inhibited Cor-1 cell proliferation; interestingly the down-regulation of cyclin D2 rather than p27kip1 provided a good molecular correlate of this. GDF11 also suppressed the expression of numerous genes implicated in cell migration, including Fascin and LIM and SH3 domain protein 1 (LASP1). In accord, GDF11 inhibited Cor-1 cell migration in a scratch wound assay. These data demonstrate GDF11 to be a master regulator of neural stem cell transcription that inhibits cell proliferation and migration by down-regulating the expression of numerous genes involved in both these processes.

## Materials and Methods

### Cell Culture

Protocols used for the derivation and culture of Cor-1 cells have been described in detail elsewhere [12], [15]. Briefly, Cor-1 cells were cultured on T75 flasks (Nunc) coated with 0.1% gelatin in PBS (Sigma) in Euromed-N media (Euroclone) supplemented with N2 (Invitrogen), L-Glutamine (2 mM, Invitrogen) as well as EGF and FGF2 (both 10 ng/ml, Peprotech), referred to in this study as “full-media”.

### Western Blotting

Cor-1 cells were cultured in 6-well plates under control conditions, or following treatments as described. Cells were lysed in 1 mM PMSF, 1 mM Na_3_VO_4_, 1 mM NaFl and 1× complete protease inhibitors (Roche) in lysis buffer (20 mM Tris-HCl pH7.4, 137 mM NaCl, 1% Triton X-100, 10% glycerol, 2 mM EDTA). Protein concentration was determined using the BCA Protein Assay Kit (Pierce). Equal concentrations of protein were separated by 7.5% SDS-PAGE and transferred to either nitrocellulose Hybond ECL membranes (Amersham) or PVDF membranes (Millipore). Membranes were blocked for 1 hr in 5% milk in Tris-buffered saline with 0.1% Tween 20 (TBS-T) and then incubated with primary antibodies that included mouse anti-ActRIIB (1∶500, Santa Cruz), rabbit anti-ALK5 (1∶500, Abcam), mouse anti-Smad 2/3 (1∶1000, BD), rabbit anti-pSmad2, pSmad3, p27kip1 and cyclin D2 (1∶1000, Cell Signalling), mouse anti-EGFR (1∶1000, Cell Signalling) or anti-beta actin (1∶10000, Abcam). After 3×10 min washes in TBS-T, membranes were incubated with horseradish peroxidase-conjugated anti-rabbit or anti-mouse IgG (1∶3000, Vector Laboratories) respectively, in TBS-T 5% milk and washed 3×10 min in TBS-T. Membranes were developed using ECL or ECL plus reagents (GE Healthcare) and exposed to X-ray film (GRI). In some instances membranes were stripped for re-probing using Re-blot Plus Strong Stripping Solution (Millipore).

### MTS Cell Proliferation Assay

100 µl of a 5×10^4 ^/ml Cor-1 cell suspension were plated into 96-well plates (Nunc) in full media containing various concentrations of GDF11. Cell number was assessed using Promega’s CellTiter 96 Aqueous cell proliferation assay kit (Promega, Southampton, UK) according to the manufacturer’s protocol and as previously described by us in detail for Cor-1 cells (Goncalves et al., 2008).

### CRE-luciferase Assay

Cor-1 cells were transiently transfected with 1 µg Luciferase reporter construct DNA and 1 µg of pRL-TK *Renilla* plasmid (Promega, Southampton, UK), using a Nucleofector II and the Cell Line Nucleofector Kit V (Lonza, Cologne, Germany) following the manufacturer’s protocol. Cells were immediately plated onto 96-well flat-bottomed plates (NUNC, Roskilde, Denmark) with the addition of fresh medium 4 and 12 hr post-seeding. Cells were treated with stated concentrations of GDF11 or vehicle 22 hr after plating. At 60 hr post-seeding cells were starved of growth factor support (EGF/FGF2) and stimulated with 10 ng/ml EGF 6 hr later, while remaining in the presence or absence of GDF11 at all times. Promoter activity was subsequently measured at 72 hr post-seeding in opaque-bottomed 96-well plates with a Veritas micoroplate luminometer (Turner Biosystems, Sunnyvale, USA) using Dual Luciferase Reporter Assay System (Promega, Southampton, UK) and following the manufacturer’s instructions. Luciferase readings were normalised for transfection efficiency by *Renilla* control values.

### Quantitative Real-Time PCR

Cor-1 cells were cultured on 0.1% gelatin-coated 10 cm dishes (Nunc) at a density of 2×10^6^ cells per dish and incubated under standard conditions. Upon reaching 80% confluency, dishes were treated with either GDF11 (25 ng/ml) or vehicle only (4 mM HCl) for 3–6 hr as indicated in the results. RNA was extracted from the cultured cells with Trizol (Invitrogen, Paisley, UK) and quantified using the Nanodrop 1000 spectrophotometer (Thermo Fisher Scientific). Trizol extracted RNA (5 µg) was reversely transcribed to cDNA using Superscript III reverse transcriptase (Invitrogen), then quantified using the Nanodrop 1000 spectrophotometer, and subsequently diluted to a concentration of 5 ng/µl with DNAse/RNAse-free water. cDNA samples were used in 25 ng reactions using SyBrGreen Mastermix (Roche Diagnostics, West Sussex, UK) in a Corbett ROTOR-gene 3000 system. RT-qPCR was performed using the following cycling conditions: 95°C for 10 min followed by 40 cycles of 95°C for 10 seconds, 60°C for 15 seconds and 72°C for 20 seconds. Finally a melt curve was produced by holding at 72°C for 10 min followed by 45 seconds hold at the first incremental step and then 5 seconds at each degree up to 95°C. Experiments were performed in triplicates. Data was analysed using the ΔΔCq method using GAPDH or HPRT as the reference gene. The primers have an efficiency of 1.00 (+/−0.20) and with sequences: Cyclin D2 Forward TCTTTCCAGAGTCATCAAGTGTG Reverse GACTCCAGAAGGGCTTCAATC; EGFRForward GCCACGCCAACTGTACCTAT Reverse GCCACACTTCACATCCTTGA; HPRT Forward CCTAAGATGAGCGCAAGTTGAA Reverse CCACAGGACTAGAACACCTGCTAA; GAPDH Forward ATACGGCTACAGCAACAGGG Reverse CCCTGTTGCTGTAGCCGTAT; Fascin Forward AGTTTGTGACCGCCAAGAAA Reverse TGAGGAAGAGTTCCGAGTCC; LASP1 Forward TGAGAAGAAGCCTTACTGCAATG Reverse CGGAGATTTTCCGGAGTG.

### Microarray Analysis

Cor-1 cells were cultured in full media in 10 cm dishes as described above and treated with GDF11 (25 ng/ml) or vehicle for 4 hr when they reached ∼70–80% confluence two days after plating. Cells were then lysed in Absolutely RNA Microprep Kit lysis buffer (Stratagene), scraped under sterile conditions, transferred to 1.5 ml eppendorf tubes and snap frozen on dry ice. RNA was then extracted from lysates and cell culture mRNA expression levels were assayed on Affymetrix mouse genome 430 2.0 arrays. In total 6 treatment and 6 vehicle control samples were generated. Probe set expression levels were obtained after normalisation with the Affymetrix MAS5.0 algorithm. The GDF11 transcriptional response was compared to previously published responses to the inhibition of canonical signalling pathways in Cor-1 cells by direct linear regression analysis of the global transcriptomes and by quantifying the overlap in significantly regulated pathways essentially as described previously [25]. The treatment response was defined by 2(t−c)/(t+c), where *t* and *c* are the average treatment and control signals. The statistical significance *p*-values for the individual probe set expression changes were calculated with a Student’s t-test and weak signals were dropped. The regression analysis was based on a simple Pearson correlation score and the statistical significance was measured by the likelihood of randomly obtaining the correlation. The full responder set is given in Table S1.

### Pathway Analysis of the Microarray Data

To identify pathways (rather than individual transcripts) that might be regulated by GDF11 we looked for enrichment of genes showing significantly altered expression in established pathway gene sets. The pathways were obtained from the Broad Institute Gene Set Enrichment Analysis (GSEA) Molecular Signatures Database (MSigDB) (www.broadinstitute.org/gsea/msigdb), consisting of 639 annotated pathways. In brief, all transcripts showing a significant (*p*<0.05) change of at least 10% were submitted for analysis against the pathway database. Pathways passing the *p*<0.01 significance threshold were considered, the significance corresponding to the probability of obtaining the given or better enrichment score with a random gene set.

### Scratch Wound Assay

COR-1 cells were plated out on Essen Image Lock 24-well plates (Essen USA) at 6×10^5 ^cells/well and kept in normal growth medium. Cells were allowed to attach at room temperature for 30 min before incubation at 37°C/8% CO_2_ to reduce clumping. Approximately 8 h after plating, GDF11 (25 ng/ml, 12.5 ng/ml or 6.25 ng/ml) or the control vehicle (4 mM HCl+0.1% BSA) were added to the cells and left overnight. Once the cells had become fully confluent (typically after 24 hr), a single uniform scratch was made along the centre of each monolayer using the Essen Woundmaker™ (Essen Instruments) to create a cell-free wound ∼900 µm wide. The wells were then washed twice with PBS to remove cell debris. Growth medium containing GDF11 or vehicle was added to the cells immediately before filming. Three pre-determined points along each scratch were imaged using the Incucyte automated imaging platform (Essen Instruments) every 2 hr for 24 hr [24]. The area of the wound infiltrated by migrating cells at 12 hr was calculated by the Essen Incucyte software. The rate of migration was obtained by measuring the area under the curve representing the change in wound width over time. The statistical significance of the results was evaluated using ANOVA.

### Quantification and Statistical Analysis

Western blots were scanned at a resolution of 2400 dots per inch using an Epson Perfection V700 Photo flatbed scanner. Band intensities were then quantified with NIH ImageJ software. Results for western blotting experiments are expressed as the means ± standard error of the mean (SEM) from a minimum of 3 experiments. A Student's t-test was used to determine differences between data groups, where *p*<0.001 (***), *p*<0.01 (**) and *p*<0.05 (*) were considered significant.

## Results

### Cor-1 cells Express Receptors for GDF11 and Respond Accordingly

**Figure 1:**
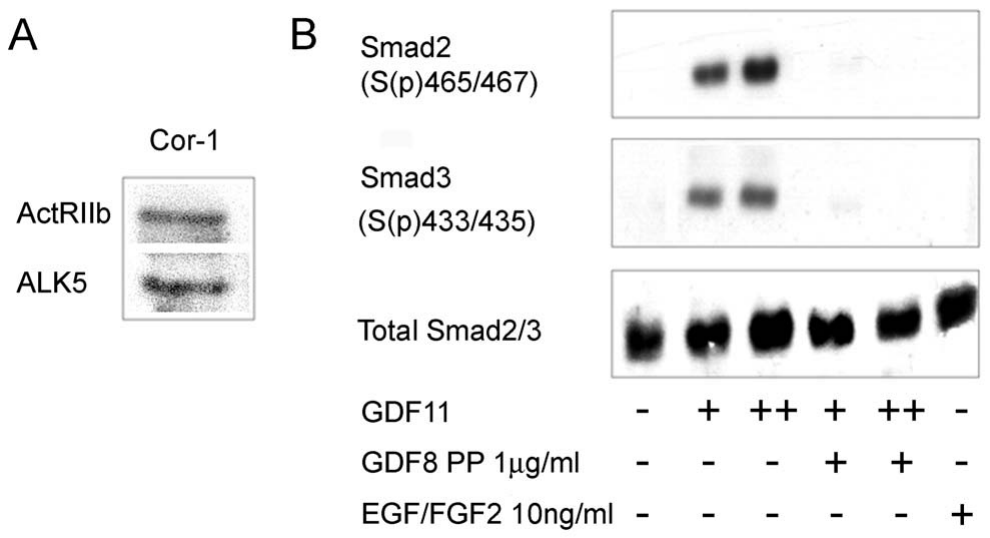
Cor-1 cells express the ALK5/ActRIIb receptor complex and respond appropriately to GDF11. (**A**) Cor-1 cells were grown until near confluence. Lysates were analysed for the presence of the ActRIIb and ALK5 receptors by western blotting. Bands at the appropriate molecular weights (70 kDa and 56 kDa, respectively) were readily detected. (**B**) Cor-1 cells were treated as indicated with GDF11 (+: 10 ng/ml, ++: 25 ng/ml) with or without the GDF8 propeptide (PP) (1 µg/ml). Lysates were probed by western blot to detect phospo-Smad2, phospho-Smad3 and total Smad2/3. EGF/FGF2 stimulation was used as a negative control.

GDF11 can signal through an ActRIIA or ActRIIB receptor generally working in partnership with the type I ALK5 receptor signalling complex (see Introduction). Our analysis of the transcriptome of Cor-1 cells pointed to the expression of transcripts for ActRIIB and ALK5 (not shown) and we used western blotting to confirm expression of both at the protein level (Fig. 1A). Canonical signalling via these receptors is initiated by the recruitment and phosphorylation of Smad proteins – with Smad2 and Smad3 being characterised downstream signalling molecules in the GDF8/11 pathway [10]. To determine if Cor-1 cells are responsive to GDF11 they were treated with 10 or 25 ng/ml of the growth factor for 2 hr before being lysed and the phosphorylation status of Smad2 and Smad3 determined using antibodies recognising phosphorylation at serine residues 465 and 467 (Smad2) and serine 433 and 435 (Smad3). Phosphorylation of both Smad proteins was readily detected following treatment with GDF11 (Fig. 1B). The GDF8 propeptide binds to GDF8 and thereby antagonises function by inhibiting growth factor binding to cellular receptors [26]. As the mature chains of GDF8 and GDF11 are virtually identical and can bind to the same receptors, the GDF8 propeptide would be expected to inhibit GDF11 function. Indeed, when present at 1 µM the propeptide fully inhibited the GDF11-induced phosphorylation of Smad2 and Smad3 (Fig. 1B). As a control, incubation with EGF and FGF did not phosphorylate Smad2/3. These observations show that Cor- are able to respond to GDF11 stimulation by Smad2/3 phosphorylation.

### GDF11 Effects on Gene Transcription

**Figure 2:**
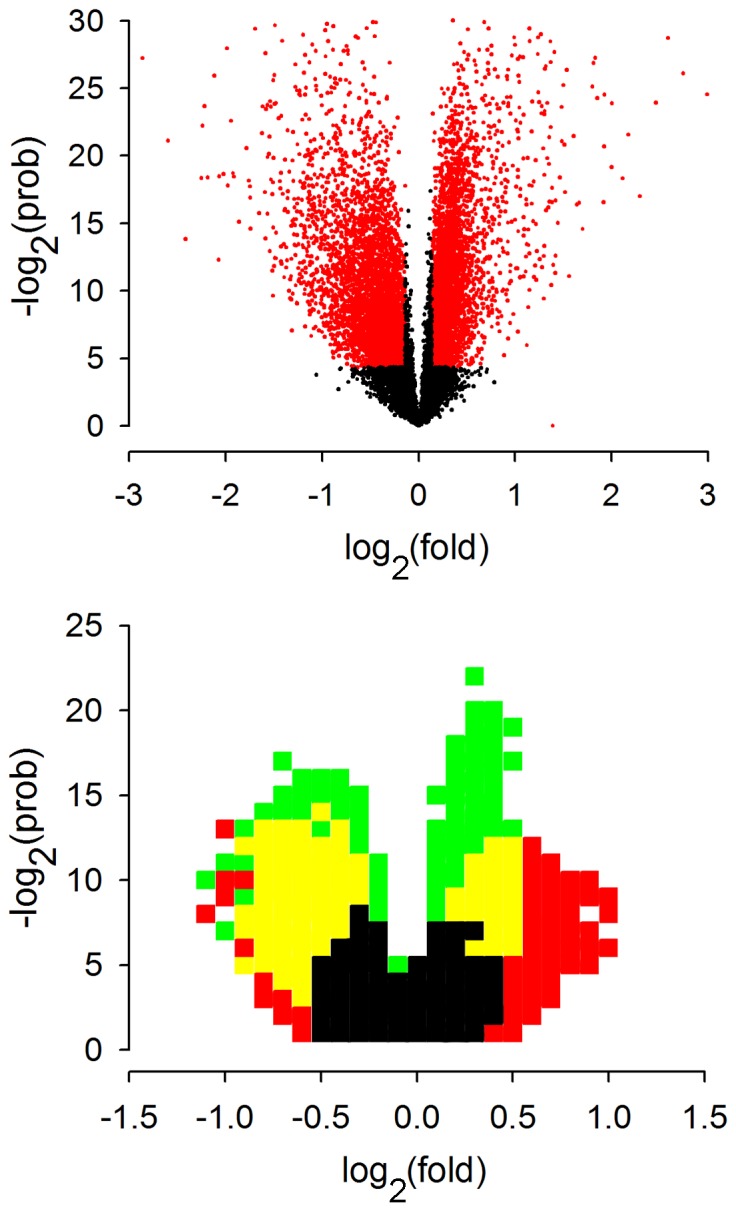
Volcano plots of the transcriptional responses to GDF11. Volcano plots are scatter plots of the log_2_ fold change against the negative log_2_ probability calculated by Student’s t-test. The points in red correspond to those probe sets passing the p<0.05 significance and 10% fold change thresholds. GDF11 treatment clearly elicits a substantial response that is skewed towards a downregulation of transcript levels (top). The relative transcriptional response to GDF11 can be compared to the other canonical pathways by superimposing the volcano plots (bottom). Here, pixels (δlog_2_(prob) = 1, δlog_2_(fold) = 0.1) are coloured if they contain more than 10 points. The ‘density plot’ illustrates the similar magnitude of GDF11 treatment (green/yellow) and EGF receptor inhibition (red,yellow), which both dominate the FGF receptor inhibition response (black).

To determine the impact of GDF11 on gene expression, we cultured Cor-1 cells in full growth media and added GDF11 (25 ng/ml) for 4 hr when cells were still in the growth phase (∼70–80% confluent). Cells were then harvested for microarray analysis. In a first level analysis we simply examined the magnitude and significance of the transcriptional response, with raw data results shown in Table S1. 8346 probes, corresponding to 4694 gene transcripts, showed a significant (*p*<0.05) change in binding with just over 22% of these changing by 50% or more; this is illustrated as a volcano plot in Fig. 2 (top). Under essentially the same conditions the EGF receptor has been shown to regulate ∼3500 transcripts, with the FGFR and eCB receptors each regulating approximately 700 transcripts [25] with this illustrated in a simple density plot (Fig. 2, bottom). The interesting point here is that whereas the EGF, FGF and eCB receptor responses are highly symmetrical, the GDF11 response is skewed towards suppression of transcription with ∼75% of the transcripts being down-regulated at the 50% fold level. Thus GDF11 can exert a dominant effect on gene expression, with a bias towards a suppression of transcription.

### Overlap in Pathways Regulated by GDF11 and other Key Receptors

**Figure 3:**
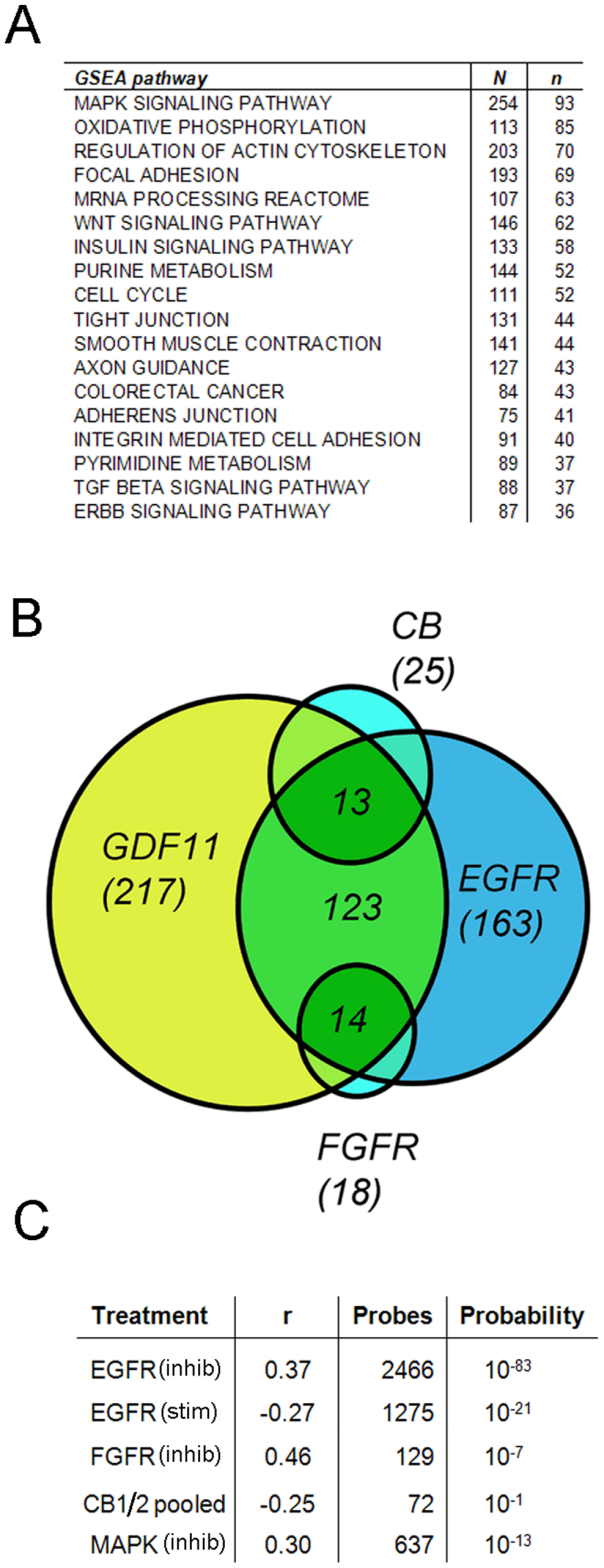
Pathway enrichment analysis for the GDF11 transcriptional response. Sets of genes whose expression was significantly altered by GDF11 treatment were scored for enrichment against the GSEA MSigDB pathway gene sets. Responder sets were considered if including transcripts with significant (p<0.05) expression changes of more than 10%. Enrichment significance was defined as the probability of obtaining the same or better enrichment score with a random set of genes. 18 of the pathways that are regulated by GDF11 are shown in (**A**) with “N” giving the number of genes assigned to the pathway and “n” showing the number regulated by the treatment. (**B**) Overlap between the pathways regulated by GDF11 and the EGF, FGF and cannabinoid receptors (CB) is shown as a simple Venn diagram. We next determined if the transcripts that are co-regulated by GDF11 and the EGF, FGF and cannabinoid receptors, or the MAPK pathway, are regulated in the same or opposite direction by determining the Pearson co-efficient *r* and statistical significance of the regression analysis as indicated in (**C**). Here, EGF receptor responders were identified by use of selective EGF receptor inhibitors (EGFR-inhib), or by direct stimulation of starved cells with EGF (EGFR-stim). See text for details.

We next identified pathways that might be regulated at the transcriptional level by GDF11 by probing all transcripts that show a significant (*p*<0.05) change of more than 10% against the Broad Institute MSigDB canonical pathway gene sets (www.broadinstitute.org/gsea/msigdb) comprising 639 pathways, and again compared this with the pathways regulated by the EGF, FGF and eCB receptors. We initially focused on a simple “quantitative” analysis with a more detailed analysis of some of the pathways considered later. When a *p*<0.01 significance threshold was applied for our comparison, GDF11 was seen to regulate 217 pathways and this was greater than the number previously shown to be regulated by the EGFR (163), the FGFR (18) or the eCB receptors (25) [25]. Of interest is the fact that ∼75% of the pathways regulated by the EGF and FGF receptors, and ∼50% of those regulated by eCB receptors, are co-regulated by GDF11. However, there are also pathways that appear to be exclusively regulated by GDF11 (summarised in Fig. 3).

### Correlations between Transcriptional Responses

The above results suggest that the GDF11, EGF, FGF and eCB receptors co-regulate a substantial number of pathways, and this might reflect regulation of individual transcripts in the same or the opposite direction. To address this question we have identified the common probe sets that are significantly (*p*<0.05) regulated by at least 25% by GDF11 and each of the above receptors and measured the Pearson correlation (*r*) between them. Here, −1≤ *r* ≤1, where a positive value is indicative of changes in the same direction and a negative value indicates co-regulation but in the opposite direction (see methods for details). A comparison of the effects of treating essentially the same cultures (maintained in media with saturating levels of EGF and FGF2) for 4 hr with GDF11, with the previously reported [25] effects of two independent selective EGF receptor inhibitors (AG1478 or PD168393 both at 100 nM), an FGF receptor inhibitor (PD173074, 500 nM) or a MAPK inhibitor (PD980059 20 mM) is summarised in Fig. 3C. GDF11 and the EGF receptor inhibitor AG1478 regulated a common set of 2466 probes, with a highly significant (*p*<10^−83^) *r* value of 0.37. A similar positive correlation was found between GDF11 and a second EGF receptor inhibitor (1536 common probes having an *r* value of 0.32, *p*<10^−37^), and although a much smaller common responder probe set was identified for GDF11 and the FGF receptor inhibitor PD173074, there was again a very highly significant positive correlation (129 common probes having an *r* value of 0.46, *p*<10^−7^). The EGF and FGF receptors synergistically regulate the transcription of a large number of genes via the MAPK pathway in Cor-1 cells [25] and there was again a very highly significant positive correlation between GDF11 treatment and treatment with a MAPK inhibitor (637 common probes having an *r* value of 0.30, *p*<10^−13^). These data demonstrate that, for a large number of genes, treatment with GDF11 has the same effect as inhibiting EGF, FGF, or the MAPK pathway. Thus GDF11 appears to regulate the same transcripts as EGF and FGF, but in opposing directions. To further evaluate this we compared the probe sets regulated by GDF11 with those regulated by treatment of starved Cor- with EGF (10 ng/ml for 3 hr). This again identified a highly significant *p*<10^−21^ regulation of 1275 common probes, but in this instance the Pearson value was negative (−0.27) confirming regulation in opposing directions.

### GDF11 Directly Suppresses Expression and Mutes Signalling via the EGF Receptor

**Figure 4:**
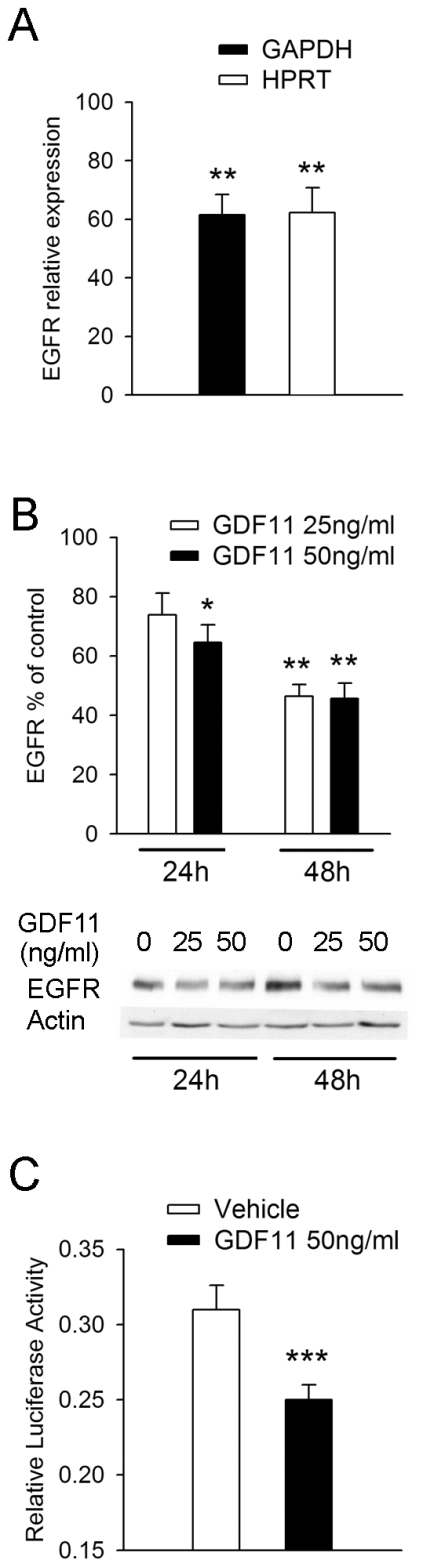
GDF11 regulates the EGF signal transduction pathway. (**A**) Q-PCR analysis of EGFR transcript levels in Cor-1 cells incubated with either control vehicle or 25 ng/ml GDF11 for 3 hr. EGF receptor expression is normalised against two housekeeping genes, GAPDH or HPRT. GDF11 significantly downregulates EGF receptor transcript levels (mean ± SEM; **p<0.01, n = 6). (**B**) Cor-1 cells incubated with 25 or 50 ng/ml GDF11 for either 24 or 48 hr were lysed and analysed for EGFR levels by Western blot. Exposure to GDF11 significantly decreases EGF receptor protein levels as shown by densitometric analysis relative to actin (mean ± SEM; *p<0.05, **p<0.01, n = 3) and by a representative blot. (**C**) Cor-1 cells were transiently co-transfected with a luciferase reporter construct and a *Renilla* plasmid by nucleofection. Twenty hours later cells were pre-incubated with 50 ng/ml GDF11 for 36 h before being starved of EGF and FGF for 6 h to reduce endogenous pCREB levels. Cells were then challenged with 10 ng/ml EGF and luciferase expression was subsequently measured by addition of a luciferase substrate. Transfection efficiency and cell numbers were taken into account by normalising luciferase readings at 570 nm against *Renilla* activity (detected by measuring light emission at 480 nm) (mean ± SEM; ***p<0.001, n = 3).

The GDF11 and EGF receptors have opposing effects on the transcription of a large pool of common responders, and this suggests close cross-talk between them. An examination of the array data shows a substantial (2.81 fold) and significant (*p*<10^−7^) reduction in EGF receptor transcripts following treatment with GDF11. To confirm this we monitored whether GDF11 affects the level of EGF receptor transcripts by quantitative PCR (Fig. 4A). We observed a highly significant (*p*<0.01) ∼40% reduction in the level of EGF receptor transcript following treatment with 25 ng/ml of GDF11 for 3 hr. Cor-1 cells were also treated with GDF11 (25 and 50 ng/ml) for up to 48 hr and the protein level of EGF receptor was detected by western blotting. This revealed a time-dependent and highly significant (*p*<0.01) reduction of ∼60% by 48 hr (Fig. 4B). The impact of this on signalling via the EGF receptor was determined using a cAMP-response element (CRE) luciferase assay. In brief, EGF receptor stimulation results in the phosphorylation of the cAMP response element binding protein (CREB), which allows it to bind to a CRE-luciferase reporter construct. Activation of the pathway results in an increase in luciferase activity, with co-transfection of a *Renilla* luciferase construct serving as a control for transfection efficiency and cell number. Cor-1 cells established in full media (containing EGF and FGF) were treated with GDF11 (50 ng/ml) or vehicle for 36 hr. EGF and FGF were then withdrawn for the following 6 hr, and the subsequent response to EGF stimulation (10 ng/ml for 6 hr) determined in the GDF11- and vehicle-treated cultures. We detected a highly significant (*p*<0.001) suppression of signalling via the EGF receptor in the GDF11-treated cultures (Fig. 4C), supporting the fact that GDF11 is able to inhibit EGF receptor signalling.

### Effects of GDF11 on Cor-1 Cell Proliferation

**Figure 5:**
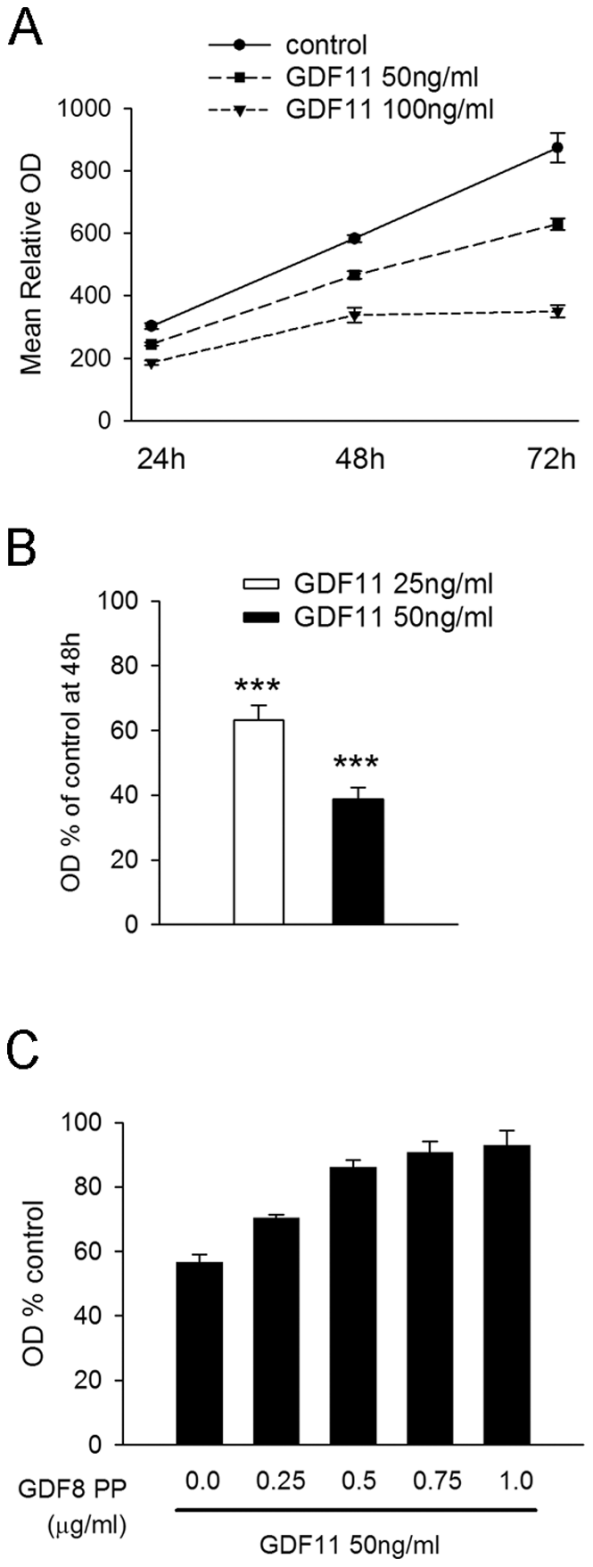
GDF11 significantly attenuates Cor-1 cell proliferation in a dose-dependent manner. Cor-1 cells were grown in 96-well plates with cell number determined relative to control cultures using the MTS assay (see Methods). A representative experiment showing GDF11-dependent inhibition of Cor-1 cell proliferation over a 72 hr period is shown in (**A**). Each data point shows the mean relative optical density (+/−SEM) from 16 replicate cultures. (**B**) Incubation with either 25 ng/ml or 50 ng/ml of GDF11 significantly inhibits Cor-1 cell proliferation as monitored by MTS assay at the 48 hr time point. Data were normalised to control values for each of 5 independent experiment (mean ± SEM; ***p<0.001, n = 5). (**C**) The inhibition of proliferation caused by GDF11 (50 ng/ml) measured at the 48 hr time point can be prevented in a dose-dependent manner by the GDF8 propeptide (PP). Shown here are results from a representative experiment, with each data point representing the mean +/− SEM relative to the control determined from 16 replicate cultures.

**Figure 6:**
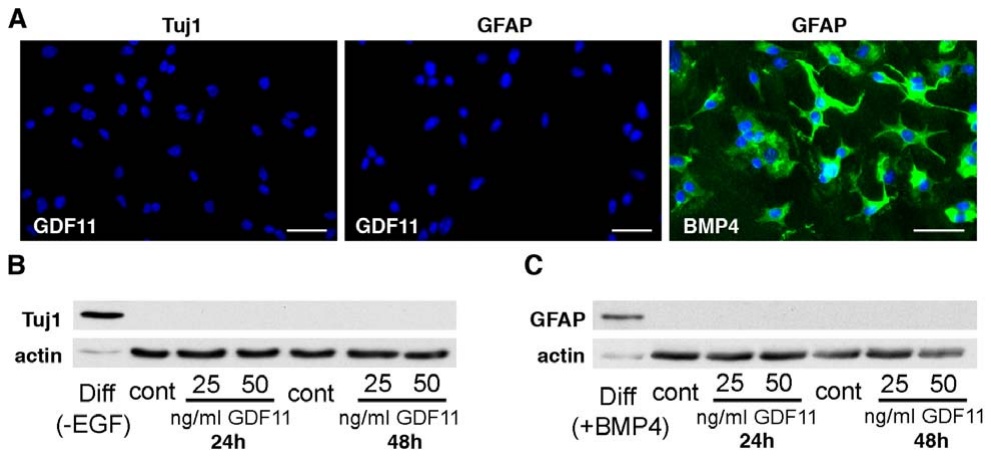
GDF11 does not induce neuronal nor astrocytic differentiation. (**A**) Cor-1 cells incubated with 50 ng/ml of GDF11 for 48 hr are negative for either the early neuronal marker Tuj1 (left) or the glial marker GFAP (centre). Incubation with 10 ng/ml of BMP4 for 48 hr induces astrocytic differentiation revealed by positive GFAP immunoreactivity. Hoechst dye (blue) was used to stain cell nuclei. Scale bars are 40 µm. Consistent with the immunocytochemistry results, Tuj1 (**B**) and GFAP (**C**) cannot be detected by Western blot in lysates of Cor-1 cells exposed to either 25 or 50 ng/ml GDF11 for 24 or 48 hr. As positive controls, Tuj1 is detected in Cor-1 cells plated on laminin and maintained for 48 hr in medium lacking EGF (**B**, first lane on the left) while GFAP is detected in Cor-1 cells undergoing astrocytic differentiation after incubation with BMP4 for 48 hr (**C**, first lane on the left).

EGF is the major mitogen for Cor-1 cells, and the standard MTS assay can be used to monitor this activity over time [15]. We cultured cells in their full growth media (containing EGF and FGF-2) and determined the effects of GDF11 (at 50–100 ng/ml) over a 72 hr period. This revealed a substantial suppression (but not complete abolition) of growth with a representative example of a time course shown in Fig. 5A. The results pooled from 5 independent experiments showed an approximate 40 and 60% reduction in cell numbers after 48 hr culture in media containing GDF11 at 25 ng/ml and 50 ng/ml respectively, relative to growth in control media (Fig. 5B). Similar responses were seen when BrdU incorporation was used to measure cell proliferation (data not shown). The response to GDF11 (at 50 ng/ml) was inhibited, in a dose-dependent manner, by the GDF8 propeptide (Fig. 5C). When tested at concentrations up to 200 ng/ml, GDF11 had no effect on the proliferation of the human embryonic kidney 293 (HEK293) cell line, the mouse embryonic NIH-3T3 fibroblast cell line or monkey COS-7 fibroblast cell line (data not shown). Thus, the inhibitory effects of GDF11 on Cor-1 cell proliferation are relatively specific. To detect whether the inhibition of proliferation was associated with an effect on differentiation, we analyzed the expression of neuronal or glial markers in GDF11-treated cells. Cor-1 cells incubated with 25 or 50 ng/ml of GDF11 for either 24 or 48 hr were negative for the neural progenitor marker Tuj1, while cells induced to differentiate towards a neuronal phenotype by plating on laminin and withdrawing EGF for 48 hr robustly expressed Tuj1 (Fig. 6A–B). Similarly, GDF11-treated cells were also negative for the astrocytic marker GFAP, while exposure to as little as 10 ng/ml of BMP4 for 48 hr induced robust GFAP expression (Fig. 6A,C).

### Effects of GDF11 on p27kip1 and Cyclin D2

**Figure 7:**
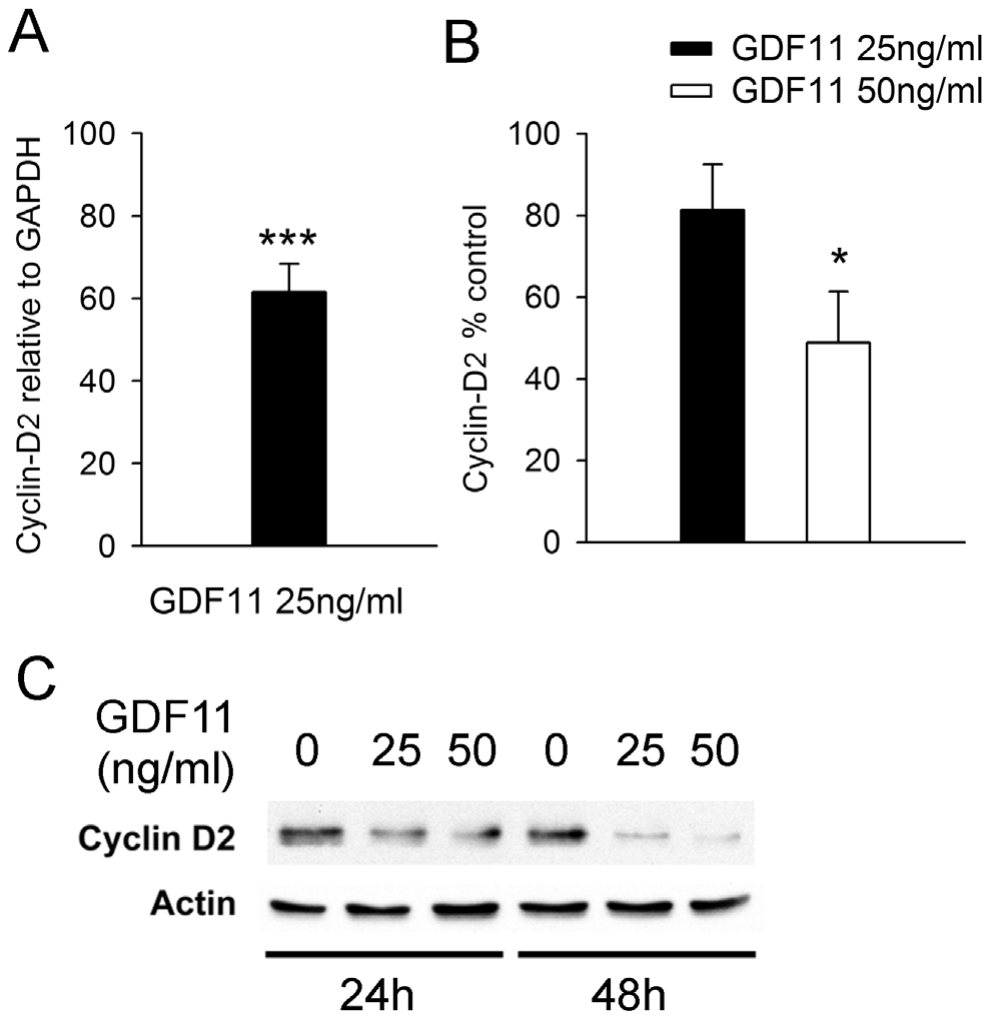
GDF11 inhibits Cyclin D2 expression. (**A**) Q-PCR analysis showing a significant decrease in Cyclin D2 levels in Cor-1 cells after treatment with 25 ng/ml of GDF11 for 3 hr. Cyclin D2 transcript levels are expressed relative to the levels of the house-keeping gene GAPDH (mean ± SEM; **p<0.01, n = 6). (**B**) Densitometric analysis of western blots of lysates from Cor-1 cells treated with 25 or 50 ng/ml of GDF11 for 24 hr shows a significant decrease in Cyclin D2 protein levels (mean ± SEM; **p<0.01, n = 3). Cyclin D2 protein levels were normalised to actin, with a representative blot probed for Cyclin D2 and actin shown in (**C**).

p27kip1 is a cyclin-dependent kinase inhibitor that suppresses cell proliferation and is regulated by several growth factors including TGFβ family members [27]. In olfactory epithelial progenitors GDF11 induces the expression of p27kip1, causing cell cycle arrest [7]. In the present study we found that GDF11 (at up to 50 ng/ml) had no effect on p27kip1 transcript or protein levels over a 48 hr treatment period (data not shown), suggesting that it regulates Cor-1 proliferation by other means. The Broad Institute MSigDB canonical pathway gene set annotates 111 “Cell-Cycle” genes with no less than 52 of these regulated by GDF11. Our eye was drawn to cyclin D2, which showed a highly significant (*p*<10^−8^) 3.6 fold reduction following GDF11 treatment, as the ratio of this to p27kip1 can regulate cell proliferation [28]. Quantitative PCR confirmed that GDF11 (a 3 hr treatment with 25 ng/ml) can significantly reduce the level of cyclin D2 transcripts in Cor-1 cells (Fig. 7A) and that this is reflected in significant loss of the protein at 24 hr (Fig. 7B–C). These data provide a molecular correlate for the effects of GDF11 on Cor-1 cell proliferation; however the inhibition of cell proliferation caused by GDF11 is unlikely to be mediated by regulation of a single transcript.

### GDF11 Suppresses NSC Migration

**Figure 8:**
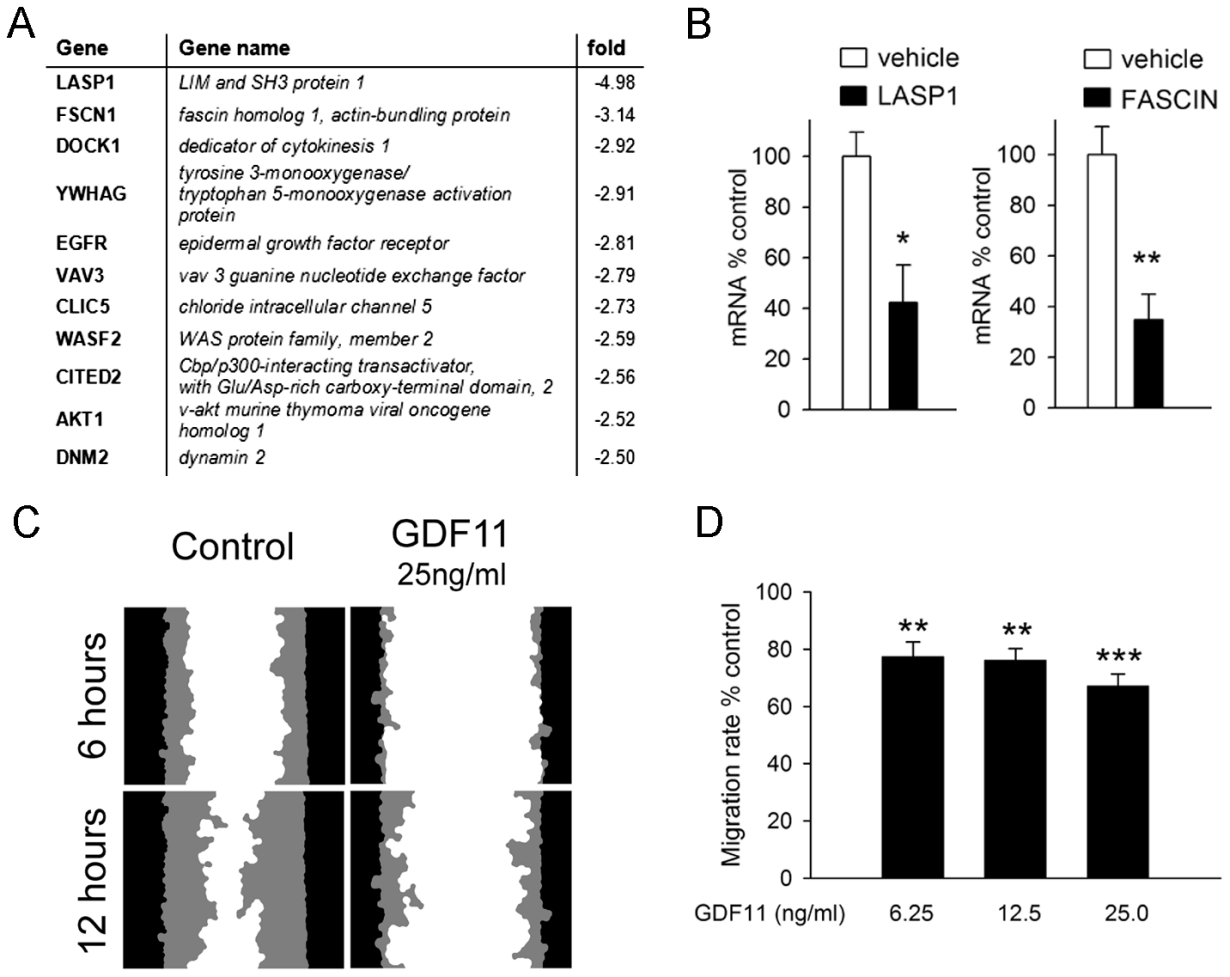
GDF11 inhibits expression of migratory transcripts and migration in Cor-1 cells. (**A**) Gene Ontology analysis of the GDF11 microarray data indicates suppression of a large number of transcripts encoding proteins associated with cell migration. Shown here are the top 11 downregulated responders. (**B**) Q-PCR analysis of Fascin and LASP1 transcript levels in Cor-1 cells incubated with either control vehicle or 25 ng/ml GDF11 for 6 hr confirmed the microarray results. Expression was normalised against GAPDH (mean ± SEM; *p<0.05; **p<0.01, n = 3). (**C**) Cor-1 cells were plated in a 24-well plate to achieve a confluent monolayer. Approximately 8 hr after plating, cells were incubated with GDF11 overnight as indicated. The following day, a wound was produced in the centre of each well and images were taken every 2 hr to monitor wound closure. Images show the initial scratch wound mask (black) and the area infiltrated by migrating cells (grey) 6 and 12 hr after scratching for control and GDF11-treated samples. (**D**) Quantification of the wound area infiltrated by migrating cells at 12 hr normalized to the control reveals a significant inhibition of migration in GDF11-treated samples (mean ± SEM; **p<0.01, ***p<0.001, n = 3).

As reported above, GDF11 suppressed the expression of 817 gene transcripts by 50% or more. In a similar way to the above pathway analysis, we looked for significantly (*p*<0.01) enriched gene ontology sets and identified 13 that are associated with cell migration (e.g. GO terms for: cell migration, cell motion, actin binding, actin cytoskeleton organisation). The list of the top 11 downregulated genes in this group is shown in Fig. 8A. We confirmed downregulation of some of these (e.g. Fascin, LASP1 and EGF receptor) by quantitative PCR (Figs. 8B and 4A). Cor-1 cell migration has been studied in detail using live cell imaging in a “scratch wound” assay [24]. This involves establishing a confluent (non-proliferative) monolayer, making a well-defined scratch in this, and monitoring closure of the wound which depends on cell migration from the scratched edge [24]. Cor-1 cells typically close the wound over 24 hr and importantly this is not inhibited by the anti-proliferative drug mitomycin C [24]. In contrast, as shown in Fig. 8C, wound closure was significantly inhibited when cells were incubated with three different concentrations of GDF11 (6.25, 12.5 and 25 ng/ml) (Fig. 8D). Together, these results show that GDF11 can suppress NSC migration, and suggest that this effect may be due to the ability of GDF11 to inhibit expression of numerous molecules associated with cell migration.

## Discussion

The stem cell niche is a highly specialised microenvironment where quiescence and expansion are in harmony. In the normal physiological state this balance allows for a continuous source of new cells for growth, maintenance or repair of a tissue. However, the optimism in the regenerative medicine field of harnessing the niche for therapeutic benefit is tempered by the possible pathological consequence of the overexuberant production of stem cells. A more complete understanding on how inhibitory and growth promoting factors interact will inform translational research in both areas.

TGFβ is generally regarded as a growth factor that suppresses cell proliferation and there is an extensive literature on how it does this by directly regulating growth arrest and also by modulating cell responsiveness to other growth factors [29]. For example, in some cells TGFβ has been shown to reduce cell responsiveness to EGF by reducing the number of high-affinity receptors [30], however in other cells TGFβ can increase the number of high affinity receptors for EGF [31]. More recently, it has been shown that whereas TGFβ1 on its own has inhibitory effects on the growth, migration and invasion of rat intestinal epithelial cells, when given with EGF it acts synergistically with the later to promote these oncogenic activities [32]. In the context of brain development, TGFβ1 has been reported to have a dominant inhibitory effect over EGF/FGF-2 signalling on neurosphere proliferation [33]. Thus, there is clearly a complex interplay between TGFβ and other growth factors – and this is magnified when one considers the large number of TGFβ family members and the diversity of receptor complexes that these ligands can engage [34].

Among the TGFβ family members, GDF11 and myostatin have redundant functions in some tissues during development, but myostatin is solely responsible for negatively regulating muscle mass [35]. GDF11 appears to have a relatively unique role in the nervous system where it can suppress neurogenesis by regulating the proliferation and competence of olfactory progenitor cells [7]–[9]. By analogy with myostatin and muscle regeneration, anti-GDF11 therapy might be of value in neurodegenerative disease; however we still require an in-depth knowledge of how this growth factor regulates neural stem cell function.

In this study we have turned our attention to the signalling pathways that might inhibit NSC proliferation. We have used the Cor-1 NSC line, which is proving itself to be useful for elucidating some of the basic biology of adult neural stem cells; for example the hypothesis for DAGL-dependent eCB signalling regulating adult neurogenesis was initially developed with Cor-1 cells before being critically tested with positive results in the adult hippocampus and SVZ using DAGLα and DAGLβ knockout mice [15], [23]. Cor-1 cells have also been used to elucidate the transcriptional basis for the cross-talk between the EGF, FGF and eCB receptors [25]. Here we show that Cor-1 cells express the GDF11 ActRIIB/ALK5 receptor complex, and more importantly that treatment with GDF11 leads to a rapid and robust activation of the canonical Smad2/3 signalling cascade. This was associated with significant changes in the expression of 4700 genes within 4 hours, with a clear skew towards a suppression of transcription. Thus, at least in terms of the magnitude of the response, the ability of the GDF11 receptor to regulate transcription is on a par with that of the EGF receptor, and substantially greater than that of the FGF or eCB receptors. Pathways regulated by EGF and FGF receptors have been identified using the Broad Institute MSigDB gene set [25] and in this study we find that 75% of these are co-regulated by GDF11. This dropped to 50% for the eCB receptors. A Pearson-based analysis of common responders showed that GDF11 directly suppresses the transcriptional responses promoted by the EGF and FGF receptors.

The transcripts regulated by the FGF receptor in Cor-1 cells are co-regulated by the EGF receptor and this can be explained by their synergistic regulation of MAPK activity in the cells [25]. It is therefore perhaps not surprising that GDF11 can suppress the transcriptional response to EGF, FGF and the MAPK pathways. This might involve cross-talk at many levels within the cell, for example pathway analysis shows that GDF11 can regulate the expression of some of the components of the MAPK pathway. However, this general effect can be explained to some extent by the observation that GDF11 regulates expression of the EGF receptor itself. Quantitative PCR confirmed a 50% reduction the level of EGF receptors transcripts a few hours after cell exposure to GDF11 and this manifests itself as a 60% reduction in receptor levels by 48 hours. Experiments with a CRE-reporter construct confirmed that this resulted in a significant suppression of EGF signalling. The EGF receptor is responsible for maintaining steady-state activation of the Akt pathway in Cor-1 cells, and this is required for cell survival [25]; however despite employing a variety of methods, including staining for the pro-apoptotic caspase 3, we failed to find any evidence for increased cell death in cultures treated with GDF11 for several days (unpublished observations). This might be accounted for by the partial, rather than full suppression of EGF receptor signalling.

Consistent with the inhibition of EGF receptor function, GDF11 suppresses Cor-1 cell proliferation. We did not detect significant modulation of lineage markers such as Mash1 or Olig2 in our microarray analysis, and GDF11-treated cells remained immunopositive for Sox2, but negative for neuronal/astrocytic lineage markers, suggesting that the inhibition of proliferation caused by GDF11 is not associated with differentiation. This is similar to what has been shown for progenitors in the olfactory epithelium, a response that is driven by an upregulation of p27kip1 [7]. We did not see any change in p27kip1 transcripts or protein in GDF11-treated Cor-1 cells; however the p27kip1/cyclin D2 ratio can also regulate cell proliferation [28]. In this context down regulation of cyclin D2 was one of the most prominent responses in the GDF11 microarray, and this was confirmed by quantitative PCR and by western blotting. Interestingly, inhibition of GDF11 signalling by follistatin substantially enhances muscle-derived stem cell proliferation. This effect was demonstrated to be in part due to the release of GDF11-dependent inhibition of cyclin D1 expression [36]. Thus, the regulation of cyclin D1/2 appears to be a general mechanism that can account for the anti-proliferative effect of GDF11, although it is worth restating that GDF11 regulates nearly 50% of the 111 transcripts that have been annotated with the “cell-cycle” label in the Broad institute gene ontology database.

During the analysis of our microarray responders, we noticed that GDF11 regulated the expression of a very large number of genes involved in cell migration. These include actin dynamics regulators like Fascin, LASP-1, LIMA1, WAVE2, Ena; microtubule plus end tracking proteins (+TIPs) such as CLASP2, EB1, EB2, NAV1; guanine nucleotide exchange factors (Vav3, RhoGEF10L, DOCK180) and GTPase activating proteins (srGAP2, ARHGAP2, SIPA1L1) modulating the activity of different Rho and Ras GTPase family members, and focal adhesion-associated proteins (paxillin, vinculin, tensin1, focal adhesion kinase). The GDF11 transcriptional response displays an overall trend towards a downregulation of pro-migratory genes (Rac GEFs, regulators of actin and microtubule dynamics) and upregulation of anti-migratory genes (Rho GEFs, stabilizers of cell-cell junction and adhesion to the extracellular matrix) in NSCs. We used quantitative PCR to validate the Fascin and LASP1 downregulation, and also demonstrated that GDF11 substantially inhibits the migration of Cor-1 cell in a scratch wound assay. The precise mechanism underlying the inhibition of Cor-1 cell migration remains to be determined – but in principle could be due to any one or a combination of the above transcriptional responses. Interestingly, a number of target genes identified by our approach have already been implicated in the control of neuroblast migration, such as the Rac GEF Vav3 [37] and members of the srGAP protein family [38]. These results provide a good list of candidates for future in vivo studies directed at determining how a non-migratory stem cell is transformed to a migratory neuroblast. Indeed, we recently demonstrated that the actin-bundling protein Fascin, one of the top downregulated GDF11 responders, is highly expressed in SVZ-derived neuroblasts and plays a crucial role for their migration *in vivo* [39].

In summary, analysis of the GDF11 transcriptional response in Cor-1 cells allows for an integrated view of the molecular changes underpinning the action of this cytokine. It is apparent that GDF11, and/or similar TGFβ family members, have the potential to exert a dominant effect on the NSC transcriptome, regulating the expression of thousands of transcripts and directly counteracting the transcriptional responses to growth-promoting factors such as EGF and FGF. These global changes most likely promote the quiescent stem cell state, or serve to limit the expansion of the transient amplifying cell. This mode of action could additionally involve maintaining cells in a non-migratory state, which in turn might prevent them leaving the stem cell niche.

## Supporting Information

Table S1 **Cor1- cell transcriptional response to GDF11 treatment.** Gene expression changes upon 4 h GDF11 treatment of Cor-1 cells. The response is shown as expression fold with associated significance statistic.
(XLS)
